# A Comprehensive Case Study of a Hyperlaxity Dilemma: An Injury-Prone Young Athlete

**DOI:** 10.7759/cureus.56245

**Published:** 2024-03-15

**Authors:** Shivansh Mehendale, Ashok M Mehendale, Avni Gakkhar

**Affiliations:** 1 Preventive Medicine, Jawaharlal Nehru Medical College, Datta Meghe Institute Of Higher Education and Research, Wardha, IND

**Keywords:** joint pain, hypermobility, beighton score, acl tear, joint hyperlaxity

## Abstract

A prevalent condition that is frequently linked to joint trauma is joint hyper-laxity. The knee joint is one of the most complex and injury-prone joints in sports. The most commonly injured is the anterior cruciate ligament (ACL). The case presented below is of a 24-year-old athlete with a past history of many sports-related injuries who is now presented with a complete tear of the ACL with hyper-laxity as a risk factor. The patient has a Beighton score of six out of nine without any other symptoms, which is suggestive of benign hyper-laxity of the joints and not hyper-laxity syndrome. Here, we emphasize that medical professionals must know the fundamental connection between hyper-laxity and musculoskeletal injuries and their proper management and rehabilitation for future prevention.

## Introduction

Joint hyper-laxity (JH) is a highly heritable disorder that causes joints to move beyond normal limits [1]. As people age, the prevalence of hyper-laxity decreases; it goes from 34% in those ages 20 to 30 to 18.4% in people ages 60 and above [2]. A 2:1 ratio is typically seen, with women experiencing a higher incidence than men [3]. Benign JH is a condition commonly observed in healthy adults without complaints. JH is also a feature of a rare, inherited, and more significant medical condition called Ehlers-Danlos syndrome, which is characterized by weakness of the connective tissues of the body. Joint hypermobility is commonly seen in people with Down syndrome and in people with Marfan syndrome. Distinguishing this from JH syndrome is crucial, as the latter is a recognized rheumatological illness that develops when a hypermobile joint is linked to soft tissue damage, arthralgia, or instability [4]. Hyper-laxity is diagnosed as a Beighton score of 4 or above [5]. Certain sports may draw people due to their inherent hyper-laxity because they are easier to perform. Nevertheless, there may be potential drawbacks since some sports have greater injury rates [6-7]. A modified form of the Carter-Wilkinson scoring system, known as the Beighton score, was employed to denote widespread hyper-laxity. A Beighton score is a useful study tool for demonstrating hyper-laxity. A high Beighton score does not always imply hyper-laxity, even though it is efficiently conducted clinically. There must also be signs and symptoms in order to diagnose hyper-laxity syndrome [8]. The most common symptom of JH syndrome is pain in joints and muscles. Other symptoms may include frequent joint and ligament injuries, including dislocations and sprains, joint and muscle stiffness, tiredness, clumsiness, poor balance, bladder and bowel issues, dizziness and fainting, and thin, stretchy skin. Researchers have found there may be a link between hypermobility and gastrointestinal issues such as irritable bowel syndrome (IBS). Conversely, a lower score must be acknowledged carefully since hyper-laxity might manifest as persistent joint pain in the neck, jaw, back, or shoulders-not measured by the Beighton score [9]. Medical specialists read the outcomes differently; some recognize a score of one out of nine as hyper-laxity. It is commonly accepted that a score of four or higher indicates hyper-laxity [10]. Components of the Beighton scale [6]: one point is given for each side right and left for the following components: aided dorsiflexion and hyper-extension of the fifth metacarpophalangeal joint more than 90°; aided apposition of the thumb to the flexor aspect of the forearm; aided hyper-extension of the elbow more than 10°; aided hyper-extension of the knee more than 10°; and one point for unaided forward flexion of the trunk with the knees fully extended so that the palms of the hands rest flat on the floor.

## Case presentation

**Figure 1:**
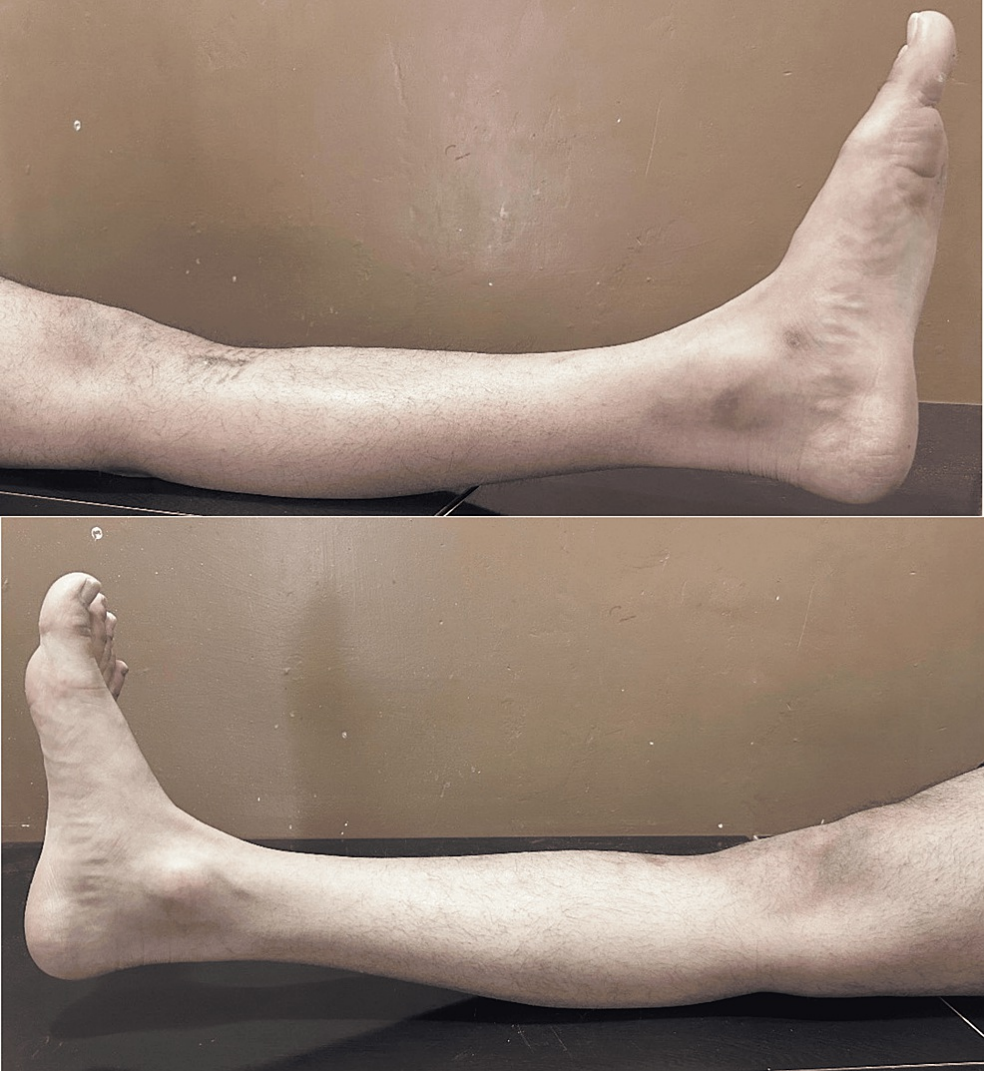
Hyper-extension of the left and right knees more than 10°

**Figure 2:**
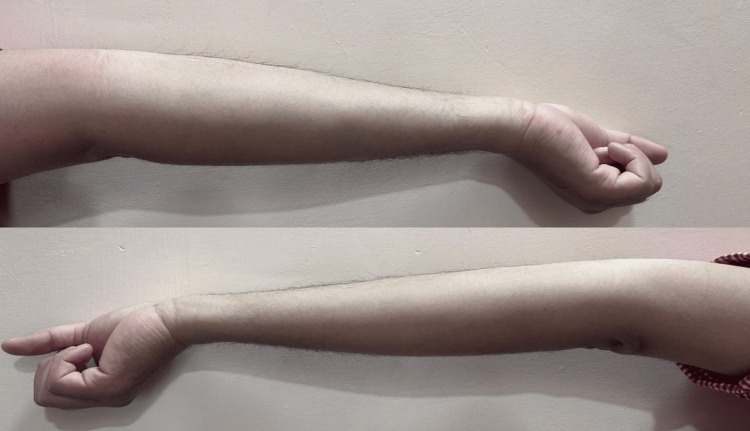
Hyper-extension of the left and right elbows more than 10°

**Figure 3:**
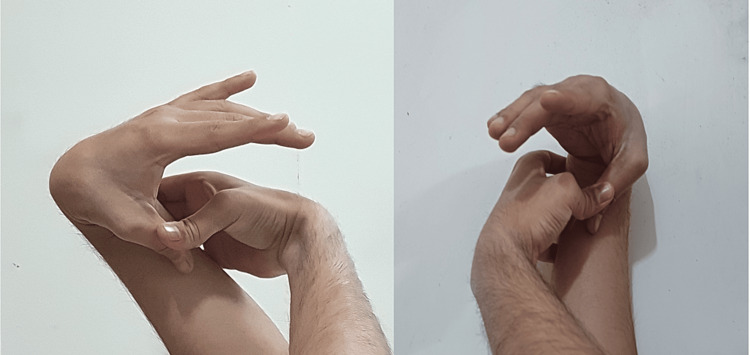
Apposition of the left and right thumbs to the flexor aspect of the forearm

A 24-year-old athlete presented with complaints of joint pain and swelling in their left knee. He sustained the injury while playing football; he landed on the hyperextended leg with a pop sound. The characteristic giveaway feeling in the left leg was present. Clinically, the anterior drawer test and the Lachman test were positive. The Thessaly and McMurray tests were negative. The patient had a Beighton score of 6 out of 9, which is shown in the following figures: Figure 1: hyper-extension of the left and right knee more than 10°; Figure 2: hyper-extension of the left and right elbow more than 10°; and Figure 3: apposition of the left and right thumb to the flexor aspect of the forearm. The patient had no joint and muscle stiffness, tiredness, clumsiness, poor balance, IBS, dizziness or fainting, thin, stretchy skin, or other complaints suggestive of connective tissue disorder. An X-ray was done, suggesting no abnormality; an MRI was done after one month when the swelling subsided; it was suggestive of a patchy marrow contusion involving the bilateral femoral condyle and posterior aspect of the medial and lateral femoral condyle. Moderate joint effusion with suprapatellar extension was seen; a complete rupture of the anterior cruciate ligament (ACL) with a surrounding heterogenous hyperintense signal was seen; and approximately 9.2 mm of the anterior fibers of the ACL were seen. The buckling of the posterior cruciate ligament was seen to be secondary to an ACL tear. The history of sports injuries was a partial tear of the anterior horn of the left lateral meniscus, a partial tear of the anteromedial fibers of the left vastus medialis with intramuscular hematoma, and a partial tear of the deltoid ligament of the left foot, which was managed conservatively a few years ago. Arthroscopic-assisted ACL reconstruction using the semitendenous and gracillis tendons of the left side was done. Weight-bearing was started three days after the operation, and physiotherapy was advised for six months, followed by muscle-strengthening exercises.

## Discussion

Activities that are strenuous or repeated are regarded as aggravating factors. Managing the load is crucial for these patients. Therefore, discussing activity/sports regimens is imperative when teaching patients and their families. Joint pain and the risk of injury can be increased by overtraining joint flexibility rather than stability [4]. The risk of reinjury is slightly higher in cases of people with JH after surgery. One of the most critical aspects of managing benign JH is exercise [10]. It has been discovered that children experiencing pain related to JH can benefit from specific joint-strengthening regimens. In this case, the patient was put into an ACL reconstruction rehabilitation program initially and later into a multi-plane sport-specific plyometrics and agility programs. First and foremost, it's critical to safeguard the joint and reduce discomfort. When severe discomfort is early in rehabilitation, braces or crutches could be a temporary fix. Similarly, strapping or orthotics may be beneficial [3]. For these patients, targeted, graded exercise programs are valuable. An excellent place to start when trying to desensitize the nervous system and build resilience is with isometric exercises [3]. Strap-on weights, light weights, and open and closed kinetic chain workouts are some strengthening options. To help develop resilience in these end-range postures, it is crucial to begin loading the joint in the hyper-extensive range as the patient gets better. Exercises for the quadriceps and hamstrings that are isometric include side-lying gluteal muscle exercises, hip abductor workouts while standing, prone eccentric hamstring workouts, and supine joint control exercises. Proprioceptive and balancing exercises should also be prioritized because research has shown that they help patients with JH feel less discomfort and perform better. Ways to prevent injuries in hyper-laxity of joints: wear supportive shoes that offer stability; work with a physical therapist who is knowledgeable about hyper-laxity; strengthen your body with isometric exercises and pilates; work on stabilizing exercises that maintain space in the joints; move your body and exercise regularly; but stick to activities that don't cause pain; avoid repetitive physical activities if they cause discomfort or irritation; keep proper form and biomechanics when engaging in physical activities; try to maintain correct posture and alignment in your body, even when you're not being physically active; stay hydrated. Seek out a doctor who specializes in or is knowledgeable about hyper-laxity, and speak with your doctor about the benefits of prolotherapy and other forms of regenerative injections.

## Conclusions

Even though people with JH have an extra edge in certain sports, they are prone to injuries. An essential part of managing joint hyperlaxity is education. The Beighton score is an important and easy tool in diagnosing JH; a score of more than 4 is usually diagnostic of JH. Healthcare providers should be able to differentiate between benign JH and JH syndrome efficiently. When a patient presents with an injury, healthcare providers should identify JH and address a number of topics, such as pain management and illness education. Patients and their families should be made aware that benign JH is a non-progressive connective tissue condition that responds well to lifestyle modifications, physical activity, and joint protection. Physiotherapy and muscle strengthening afterward, along with general conditioning, are usually necessary to manage symptoms and prevent further damage. Strength and proprioception improvement are critical components of rehabilitation.
